# Inflammatory response to the administration of mesenchymal stem cells in an equine experimental model: effect of autologous, and single and repeat doses of pooled allogeneic cells in healthy joints

**DOI:** 10.1186/s12917-016-0692-x

**Published:** 2016-03-31

**Authors:** N. Ardanaz, F. J. Vázquez, A. Romero, A. R. Remacha, L. Barrachina, A. Sanz, B. Ranera, A. Vitoria, J. Albareda, M. Prades, P. Zaragoza, I. Martín-Burriel, C. Rodellar

**Affiliations:** Servicio de Cirugía y Medicina Equina, Hospital Veterinario, Universidad de Zaragoza, C/Miguel Servet, 177, Zaragoza, 50013 Spain; Laboratorio de Genética Bioquímica LAGENBIO, Universidad de Zaragoza, C/Miguel Servet, 177, Zaragoza, 50013 Spain; Departamento de Cirugía Ortopédica y Traumatología, Hospital Universitario Lozano Blesa, Universidad de Zaragoza, San Juan Bosco, 15, Zaragoza, 50009 Spain; Departament de Medicina i Cirugia Animal, Universidad Autónoma de Barcelona, Edifici H, UAB, 08193 Bellaterra, Barcelona, Spain

**Keywords:** Allogeneic, Horses, Joint, Mesenchymal stem cells, MSCs

## Abstract

**Background:**

Mesenchymal stem cells (MSCs) transplantation has become a promising therapeutic choice for musculoskeletal injuries. Joint-related disorders are highly prevalent in horses. Therefore, these animals are considered as suitable models for testing MSC-based therapies for these diseases. The aim of this study was to investigate the clinical and inflammatory responses to intra-articular single and repeat dose administration of autologous or of pooled allogeneic MSCs in healthy equine healthy joints. Six horses were intra-articularly injected with a single autologous dose of bone marrow derived MSCs (BM-MSCs) and two separate doses of allogeneic BM-MSCs pooled from several donors. All contralateral joints were injected with Lactated Ringer’s Solution (LRS) as the control vehicle. Signs of synovitis and lameness were evaluated at days 0, 1, 2, 3, 5 and 10 after injection. Total protein (TP), white blood cell count (WBC) and neutrophil count (NC) in synovial fluid were also measured at the same time-points.

**Results:**

A mild synovial effusion without associated lameness was observed after all BM-MSCs injections. The second allogeneic injection caused the lowest signs of synovitis. Local temperature slightly increased after all BM-MSCs treatments compared to the controls. TP, WBC and NC in synovial fluids also increased during days 1 to 5 after all BM-MSCs injections. Both, clinical and synovial parameters were progressively normalized and by day 10 post-inoculation appeared indistinguishable from controls.

**Conclusions:**

Intra-articular administration of an allogeneic pool of BM-MSCs represents a safe therapeutic strategy to enhance MSCs availability. Importantly, the absence of hypersensitivity response to the second allogeneic BM-MSCs injection validates the use of repeat dose treatments to potentiate the therapeutic benefit of these cells. These results notably contribute to the development of stem cell based therapies for equine and human joint diseases.

**Electronic supplementary material:**

The online version of this article (doi:10.1186/s12917-016-0692-x) contains supplementary material, which is available to authorized users.

## Background

Osteoarthritis (OA) is an irreversible degenerative disease characterized by articular cartilage loss and synovial inflammation. Current OA therapeutic strategies are focused on reducing pain, physical disability and handicap, and try to limit structural deterioration in affected joints [1, 2].

Cell therapy using stem cells has become a large field of research focusing on the development of effective treatments for this disease [3]. Mesenchymal stem cells (MSCs) are studied as a possible tool for cell therapy not only by their “stem” properties and differentiation potential but also by their trophic and immunomodulatory properties [4, 5]. Horses commonly suffer from OA and osteochondrosis, being diseases of great concern for equine clinicians [6]. In addition, horses are considered the most appropriate animal model for testing the clinical effects of MSC-based therapies for humans joint injuries [7].

While both autologous and allogeneic MSCs therapies have been used for the treatment of several diseases [8], the use of allogeneic MSCs has gained relevance due to their shorter *ex vivo* expansion time [9] and the possibility to be selected according to their characteristics to optimize the treatment (higher immunomodulatory capacity, rate of growth in culture, etc).

The administration of a single dose and repeat doses of allogeneic-derived MSCs obtained from one donor has been tested in equine under different conditions [10–12]. Although it has been demonstrated that repeat injections of MSCs can enhance the benefit of these cells in different pathology models and administration routes [13, 14], it remains unclear if allogeneic MSCs can provoke an immunoresponse [15, 16]. Using single-donor allogeneic MSCs has some constraints, such as the donor selection or the number of cells obtained under culture conditions. Therefore, the use of MSCs pooled from several donors could be advantageous.

To our knowledge, safety of repeat intra-articular administrations of allogeneic bone-marrow derived MSCs (BM-MSCs) pooled from several donors has not been yet studied in horses. Neither their safety profile can be extrapolated from allogeneic-single administration of one-donor MSCs [10, 17]. Hence, in this work we evaluate the clinical and inflammatory response to the administration of autologous and repeat doses of allogeneic BM-MSCs pooled from several donors in tarso-crural and radio-carpal equine healthy joints.

## Results

### MSC isolation, differentiation and characterization

**Fig. 1:**
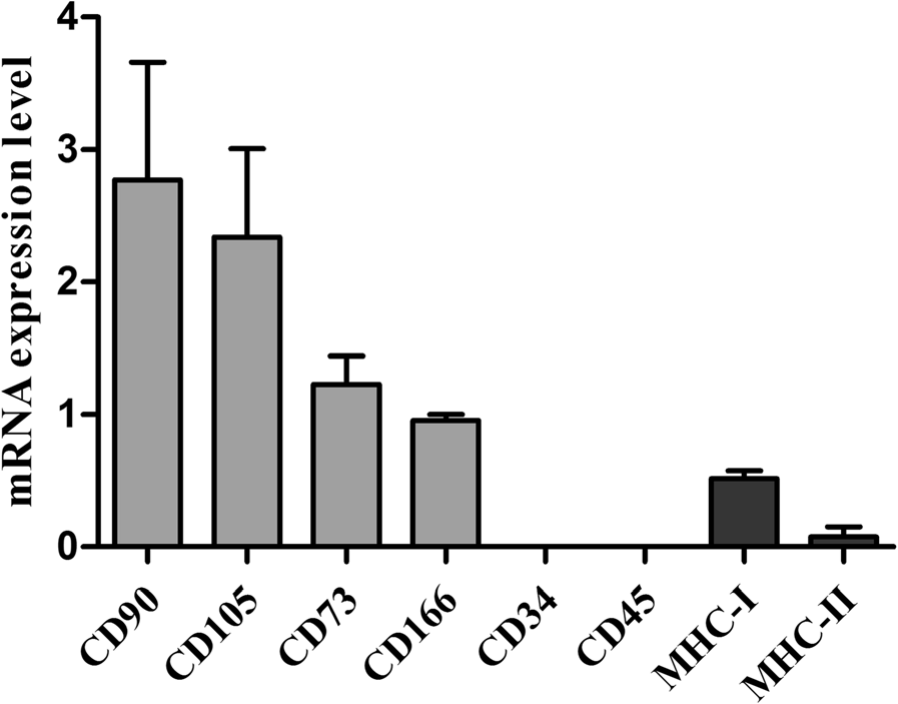
Mean ± standard error (S.E.) of relative mRNA expression (y axis) of 6 antigens surface markers (light grey) and 2 major histocompatibility complex molecules (dark grey) (x axis) for BM-MSCs (*n* = 6) examined by RT-qPCR. The BM-MSC used in this study were positive for CD90, CD105, CD73, CD166 and MHC-I and negative for CD34 and CD45. MHC-II was expressed at low level

Approximately 80 x 10^6^ BM-MSCs in third passage were successfully obtained from the bone marrow aspirate of each horse. Gene expression of the surface marker antigens *CD90*, *CD105, CD73* and *CD166* were positive, whereas no mRNA was detected for haematopoietic markers *CD45* and *CD34*. Specific dyes confirmed the ability of the cells to differentiate into osteogenic, adipogenic and chondrogenic lineages after induction with corresponding media. Characterization data are presented in Fig. 1 and Additional file 1. Viability and proliferation of cryopreserved cells was confirmed by the maintenance of similar cell doubling time (DT) after thawing. MSC gene expression of *MHC-I* and *MHC-II* is showed in Fig. 1. Moderate expression of *MHC-*I and low expression of *MHC-*II were reported, according to previous studies [18].

### Clinical assessment

**Fig. 2:**
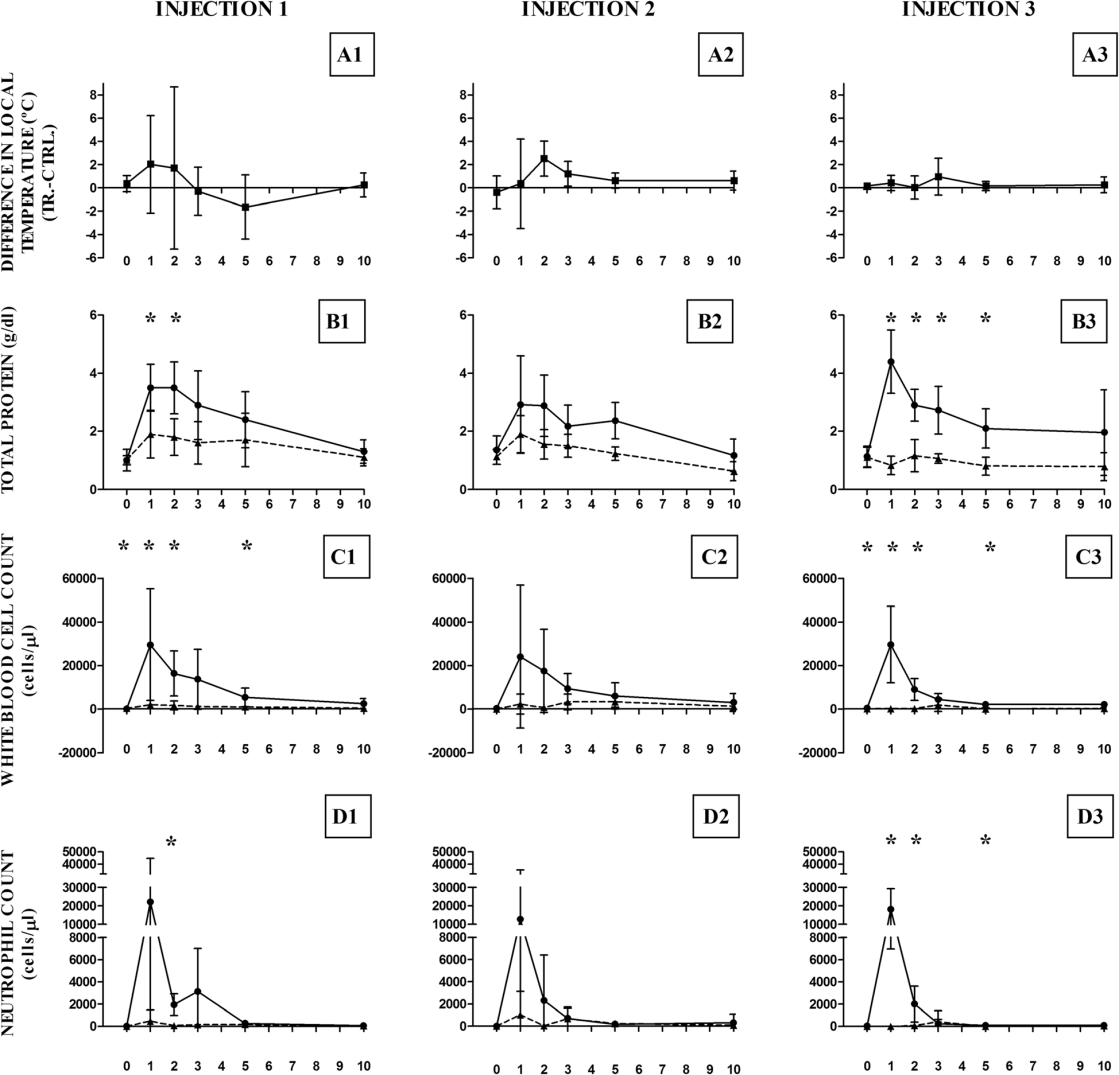
Mean ± standard deviation of difference in local temperature and synovial fluid parameters in Injections 1, 2 and 3. Mean ± standard deviation (SD) of difference in local temperature and synovial fluid parameters analyzed for autologous (Injection 1), allogeneic (Injection 2) and repeat allogeneic (Injection 3) mesenchymal stem cell (MSC) injected joints compared to controls. **a** Difference in local temperature (°C) between MSC injected joints (TR.) and control (CTRL.) joints (local temperature in MSC injected joints – local temperature in control joints) at each time point for Injection 1 (A1), Injection 2 (A2) and Injection 3 (A3). **b** Total protein concentration (g/dL) at each time point for Injection 1 (B1), Injection 2 (B2) and Injection 3 (B3). **c** White blood cell count (cells/μl) at each time point for Injection 1 (C1), Injection 2 (C2) and Injection 3 (C3). **d** Neutrophil count (cells/μl) at each time point for Injection 1 (D1), Injection 2 (D2) and Injection 3 (D3). Asterisk symbol (*) indicates statistically significant difference (*p <0.05)* between mean ± SD in control and MSC treated joints at each time point. Controls are represented as dotted lines and treated as solid lines

No visual evidence of synovitis was observed in the control joints in any of three injections. Some articular distension was detected on treated joints in all experiments. The visual exam showed a marked distension only in two joints injected with autologous MSCs (Injection 1). Mild synovial effusion after the first allogeneic pool of MSCs administration (Injection 2) was observed, whereas the allogeneic reinjection (Injection 3) provoked only a slight distention. In addition, a slight increase in local temperature was observed (Fig. 2), ranging from 0.5 to 3 °C after Injection 1 in the treated joints compared with contralateral joints injected with Lactated Ringer’s Solution (LRS) as control. In Injections 2 and 3, the average temperature increase in treated joints respect their controls was less than 1 °C. In all cases, the normal values were restored between 3 and 10 days and differences were not statistically significant at any time for any Injection.

**Fig. 3:**
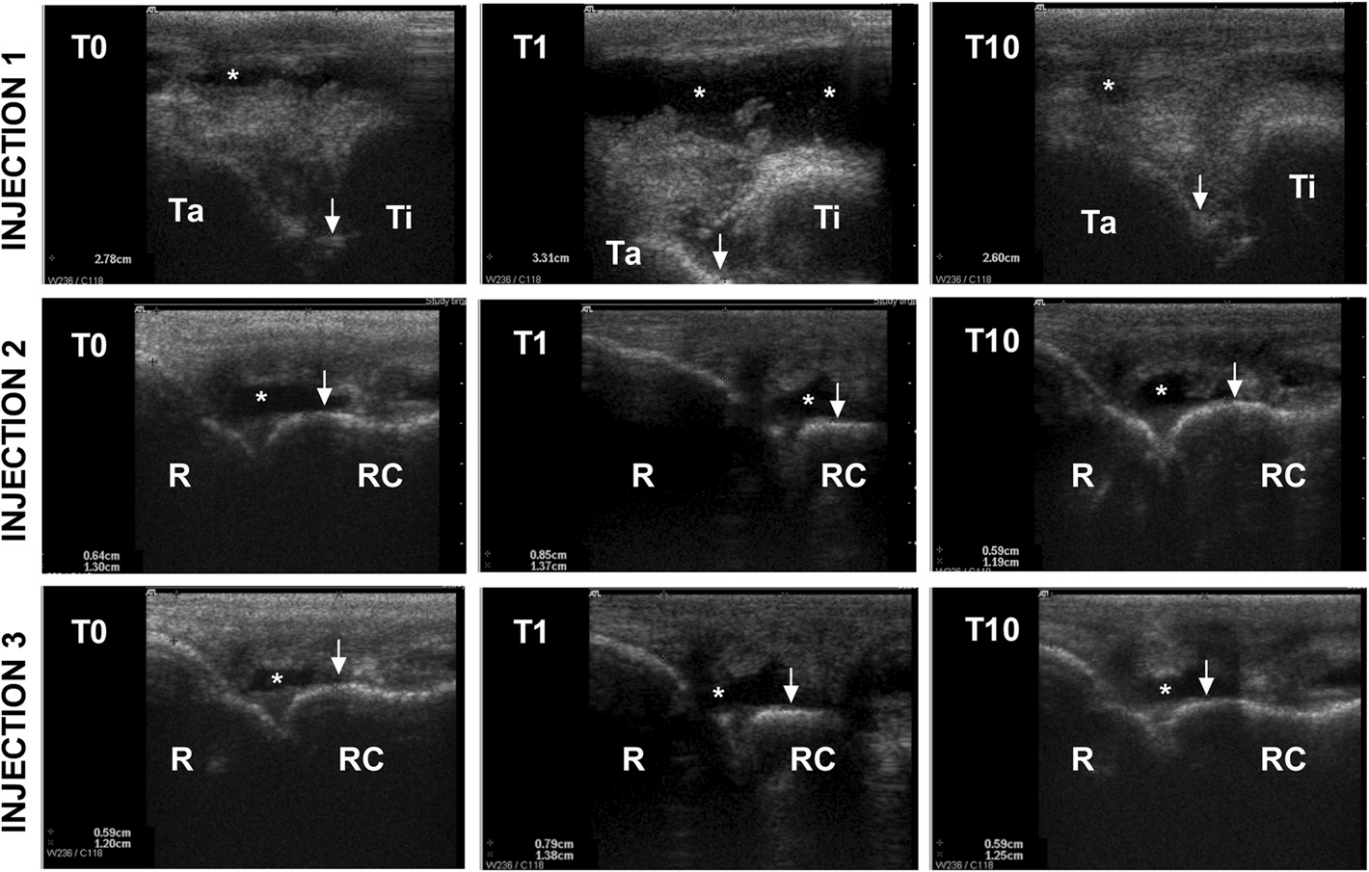
Longitudinal ultrasound images of representative MSC-injected tarso-crural and radio-carpal joints after Injections 1, 2 and 3. The transducer was longitudinally placed on the lateral plantar pouch of the tibiotarsal joint or in the dorsal aspect of the carpus, parallel and medial to the extensor carpi radialis tendon, at the level of the radio-carpal joint. Images are recorded previous to the IA injection of BM-MSCs (T0), and 1 day (T1) and 10 days (T10) after BM-MSC injection. Distance between the bone surface and the skin was measured in two points at every time. Slight distension and effusion was observed 1 day after all BM-MSC injection, which was normalized by the 10^th^ day. Ta = talus, Ti = tibia, R = radius, RC = radio-carpal bone, * = synovial fluid (effusion) in tarso-crural or radio-carpal joint, white arrow indicates the bone surface

Throughout the study none of the animals showed lameness in trot, except one horse after Injection 1. The lameness gradually decreased over time and absent at day 10 (Additional file 2). Ultrasonography findings were compatible with transient slight synovitis in treated animals receiving Injections 1, 2 and 3. Ultrasonographic signs of synovitis disappears by10 days post-inoculation. Re-injected joints (Injection 3) showed the lowest signs of synovitis (Fig. 3).

### Assessment of synovial fluids

TP, WBC and NC values at each time-point are represented in Fig. 2 and Additional file 3 .

### Total protein (TP)

A non-significant transient increase of TP values (<2gr/dL) was observed in control joints injected with LRS. Significant differences in TP values between treated and control joints were detected post-injection on days 1 and 2 (Injection 1), days 2 and 5 (Injection 2) and days 1, 2, 3 and 5 (Injection 3). The largest TP increase was detected 24 h post-Injection 3. In all cases the TP progressively decreased reaching normal-healthy values (<2gr/dL) on the 10^th^ day post-inoculation.

### White blood cells count (WBC)

A moderate rise in WBC was observed in all control joints (Additional file 3). WBC counts increased in all treated joints. The increase was statistically significant after inoculation on days 1, 2 and 5 after Injections 1 and 3. No significant differences in WBC values were found after Injection 2 at any time point. WBC values diminished to normal reference ones in all cases by the 10^th^ day.

### Neutrophil count (NC)

Control joints showed a slight increase in NC after the three Injections (Additional file 3). The NC increase in treated joints was evident on day 1 after the three Injections, being statistically significant for Injections 1 and 3. This NC increment suddenly decreased at 48 h post-injection and remained low until reaching normal reference range on day 10.

## Discussion

In our work, we have evaluated the clinical and inflammatory responses of healthy equine joints after intra-articular injections of autologous and allogeneic single and repeat pool of BM-MSCs. BM-MSCs were used due to their regenerative performance and its ability to integrate into the soft tissue joint [19, 20] as well as their anti-inflammatory and immunomodulatory properties [4]. Equine species was selected due to its availability as human joint disease model [21] and its own relevance in veterinary medicine [22]. Healthy joints were chosen to approach articular immunoresponse to repeat allogeneic injections of BM-MSCs. Behavior and properties of MSCs, such their migratory pattern [23] or the safety of therapeutic strategies based on MSCs [24, 25], are usually prior studied in healthy animals. The conditions in healthy and inflamed joints are different (i.e. vascularization, leukocyte traffic), so the response of healthy joints to repeat allogeneic MSCs cannot be directly extrapolated. Despite these differences, performing a preliminary study in healthy joints seems mandatory since if a negative response in normal joints would have been observed, this would discourage the use of repeat allogeneic doses of MSCs in altered joints.

There are a few of very recent studies comparing the immune response to autologous and allogeneic mesenchymal stem cells intra-articular injections in horses, both in normal joints [10, 26, 27] and inflamed joints [28]. However, none of these studies assess the use of a pool of allogeneic donors and the re-inoculation of joints with a second dose of cells. The use of repeat doses of MSCs could be very important in order to potentiate the MSC therapeutic effect, as it has been demonstrated in other species and diseases [13, 14]. Using a pool of allogeneic MSC donors is also interesting in order to avoid individual variations and limited number of MSCs in some cases.

The BM-MSCs showed a normal growth pattern, with a proliferation rate and viability similar to other studies [29, 30]. After cryopreservation, the cells showed a normal growth pattern indicating no effect on growth.

According to previous reports [31], equine BM-MSCs displayed similar gene expression of the surface markers. MSCs used in our study were in passage 3 to avoid the risk of morphological or functional changes that might occur at long-term culture [10].

Intra-articular injections were performed using autologous and allogeneic pools of BM-MSCs. Allogeneic MSC therapy is an interesting strategy using “off-the-shelf” product. Allogeneic therapy avoid autologous limitations such as the scarcity of MSCs, especially in cases where MSC are low and the quality might be compromised as it happens in aged individuals and disease, and also in situations where autologous MSCs might have the same genetic defects as the patient [9, 32]. In addition, allogeneic use of MSCs offers the possibility of selecting the most suitable donors according to their proliferation rate, their differentiation ability or their immunoregulatory properties.

The allogeneic BM-MSC pool offers the advantage of availability of elevated number of MSCs for the treatment of acute orthopedic lesions without the inherent period (4–6 weeks) associated with isolation and expansion of autologous MSCs [26]. Furthermore, the proliferation rate and chondrogenic differentiation capacity of MSCs might be different because of individual-related factors [11] and also their immunomodulatory properties, so the use of several donors could attenuate this variability. The results of this study show that intra-articular inoculation of equine BM-MSC, independently of their origin (autologous or allogeneic pool), or the number of doses, produces a transitory inflammatory reaction that becomes restored to normal values 10 days later, without any anti-inflammatory or analgesic drug administration. This temporary inflammation was detected in both clinical and synovial (TP, WBC and NC) parameters. Our results are similar to other clinical evidence recently described after injection of autologous and allogeneic MSCs in equine healthy joints [10, 11]. Similarly, reports using an equine synovitis model have showed that allogeneic MSCs induce an initial mild self-limiting inflammatory reaction when administered within the inflamed joint, but MSCs reduced the synovial fluid cell populations in this model [28]. Slight lameness was noted only in one animal 24 h post autologous MSC administration (Injection 1). This animal also showed more marked signs of synovitis at this time. Allogeneic MSCs injection did not lead to any grade of lameness in any horse at any time. Mild to severe lameness 24 h post injection of allogeneic CB-MSCs in inflamed joints has been reported, but lameness did not correlate with synovitis induced by MSCs injection [28].

We assessed the articular inflammatory response after a second inoculation of the allogeneic equine MSC (Injection 3). No hypersensitivity response was observed. The re-injection caused a transient inflammatory reaction similar to that detected after Injections 1 and 2. Synovial parameters (TP, WBC and NC) changes were more evident than clinical signs, but spontaneously returned to baseline by 10 days in all cases.

Although the cause of inflammation produced by the injection of BM-MSCs is still unknown [26], there are different factors that might be taken into account. The production, expansion and infiltration procedures were carried out in aseptic conditions, leaving out a possible septic origin that was supported by no detection of clinical or cytological evidence of a possible septic arthritis. Besides the inflammation was self-limiting and resolved without any medication, which is incompatible with the development of iatrogenic joint sepsis [33]. There is controversy about the cause of this joint inflammation. Some authors point out that any articular injection may cause at least a slight synovitis, or even a kind of reactive synovitis could occur after joint injection of any type of product [33–35]. Other authors suggest that the inflammatory response that follows administration of MSCs may be due to the exquisite sensitivity of healthy equine joints to the introduction of pro-inflammatory cytokines that may be present in cell culture, or even to chemical or biologically active substances present in the culture medium used, such as fetal bovine serum [26]. Recently, it has been demonstrated that equine allogeneic MSCs are capable of eliciting antibody responses in vivo, which might be cross-reactive with donor MHC types different to the recipient [36]. In our experimental conditions, our results suggest that the significant increase in synovial parameters detected during the first few days after LRS injection could be an inflammatory effect of repeated arthrocentesis. Similar results were found by Jacobsen et.al*.* [37] and Sanchez Teran et.al. [38].

Differential inflammatory responsiveness of the joints have also been discussed [11]. Indeed, the distal interphalangeal joint and tarso-crural joint are more susceptible to reactive arthritis following intra-articular injection [37]. In this context, differences in the inflammatory response between autologous BM-MSCs and the allogeneic pool of BM-MSC treated joints could be due to inter-individual and inter-joint variability. Certainly, the autologous injection was made in the tarso-crural joint and allogeneic doses were administered in the radio-carpal joint, which seems to be less prone to inflammatory reaction than the tarso-crural joint [34].

In agreement with other similar trials, our results showed higher WBC and NC increases than those observed for protein values [10, 26]. This difference may be due to the kinetics of these two inflammation markers [26, 39]. This trend has also been observed in other works analyzing the response of equine synovial fluid to intra-articular injection of different inflammatory stimuli [40].

## Conclusion

Our experiments have shown a transitory inflammatory response in all injected equine healthy joints to the administration of autologous, and single and repeat doses of allogeneic MSCs pooled from several donors. This situation resolved spontaneously within 10 days post-inoculation. The different conditions in healthy and inflamed joints do not allow to directly extrapolating our results to an articular damage situation. Despite this, we suggest based on these findings that repeat intra-articular administration of an allogeneic pool of BM-MSCs could be used as a safe strategy, enhancing the MSCs availability. The absence of a hypersensitivity response to the second allogeneic BM-MSCs injection could also be considered an important contribution to the in vivo transplantation of MSCs, since several injections could potentiate the therapeutic benefit of these cells. These results could notably contribute to the development of stem cell based therapies for equine and human joint diseases.

## Methods

### Animals

Six crossbreed saddle gelding horses aged from 3 to 7 years were used in this study. Absence of musculoskeletal abnormalities in the tarso-crural and radio-carpal joints was determined by clinical and radiological examination, including absence of lameness, palpation, range of motion, static and dynamic flexion tolerance and absence of radiological abnormalities.

All procedures were carried out under Project Licence (PI 09/12) approved by the in-house Ethic Committee for Animal Experiments from the University of Zaragoza. The care and use of animals were performed in accordance with the Spanish Policy for Animal Protection RD53/2013, which meets the European Union Directive 2010/63 on the protection of animals used for experimental and other scientific purposes.

### Study design

Three batches of injections (Injections 1, 2 and 3) were performed in all horses by a single blinded researcher (Nekane Ardanaz: NA). Joint treatments were randomly assigned. The same researcher also carried out the assessment of the clinical and inflammatory response on days 0, 1, 2, 3, 5 and 10 after each intra-articular administration of the tree Injections. Horses were not bandaged or medicated with any anti-inflammatory, antibiotic or analgesic drugs in order to not interfere with the inflammatory response, and to provide an unaltered clinical response to the MSC injections throughout the experiment.

- **Injection 1:** All horses were blindly injected in the tarso-crural joints. One of the joints was inoculated with 3 mL of Lactated Ringer’s solution (LRS), which was used as control vehicle. The contralateral joint was treated with 25x10^6^ autologous BM-MSCs suspended in 3 mL of LRS.
- **Injection 2:** Ten days after the Injection 1 each horse was blindly inoculated in their radio-carpal joints. One of the joints was injected with 3 mL of LRS (control) and the contralateral was inoculated with a pool of 25x10^6^ allogeneic BM-MSCs (5x10^6^ BM-MSCs from each donor) diluted in 3 mL of LRS. For each individual horse, the BM-MSC pool used was derived from all other animals in the study but excluded BM-MSCs from the animal in question.
- **Injection 3:** The same procedure described for Injection 2 was replicated 10 days later, once the inflammatory parameters after Injection 2 returned to normality.

### MSC isolation, culture, expansion, cryopreservation, differentiation and characterization

Horses were sedated with romifidine (0.04 mg/kg IV) and butorphanol (0.02 mg/kg IV) and were placed in a restrain stock. Approximately 60 mL of bone marrow (BM) aspirate from the 5^th^ sternebrae from each horse were aseptically collected in heparinized syringes [41]. Bone marrow derived mesenchymal stem cells (BM-MSCs) were obtained from the BM aspirates.

BM aspirates were diluted 1:3 with PBS and then layered over Lymphoprep (Atom) and centrifuged for 20 min at 1700 g. The mononucleated cell population layer above the Lymphoprep was aspirated and washed twice with PBS (Gibco). The pellet was resuspended in 10 mL growth medium, consisting of low glucose Dulbecco Modified Eagle’s Medium (DMEM) and supplemented with 10 % fetal bovine serum, 1 % glutamine and 1 % streptomycin/penicillin (all from Sigma-Aldrich). Cells were counted, plated at a density of 2 × 10^6^ nucleated cells/cm^2^ in 6-well plates and incubated at 37 °C, 5 % CO_2_. Cells were washed twice with PBS after 24, 48 and 72 h of incubation and were maintained in growth medium until reaching approximately 80 % confluence. The cells were then detached by treating with 0.25 % trypsin-EDTA (Sigma Aldrich), counted in a Neubauer counting chamber using trypan blue staining, and plated in T75 or T175 flasks (Becton Dickinson) at 5000 cells/cm^2^. The cells were trypsinised repeatedly until the third passage and then were cryopreserved in 10^6^ aliquots with freezing medium consisting on 90 % FBS and 10 % DMSO. Approximately 10^6^ cells from passage three were thawed at 37 °C and plated in a T75 flask for three days to re-adjust the culture conditions prior to being characterized and used on the different Injections.

Cells were characterized as BM-MSCs by tri-lineage differentiation and gene expression profile of cell surface markers. The tri-lineage differentiation ability was assessed plating cells at 20.000 cells/cm^2^ or 2.500 cells/cm^2^ in 12-well plates and cultured for 7 or 14 days in osteogenic or adipogenic medium, respectively. For chondrogenic differentiation, approximately 500.000 cells were pelleted in 15 mL conical tubes and cultured in chondrogenic medium for 21 days. Differentiation ability was evaluated for each lineage by specific staining, using Alizarin Red S (Sigma-Aldrich), Oil Red O and Alcian Blue for osteogenic, adipogenic and chondrogenic differentiation, respectively. Methodology and composition of differentiation media were described by Ranera et al [31].

Phenotype of the BM-MSCs was determined by Real Time quantitative polymerase chain reaction (RT-qPCR). Total RNA was isolated from approximately 10^6^ cells using the RNA spin mini (GE Healthcare Lifesciences, LittleChalfont, UK) and DNAse turbo (Ambion, Foster City, California, USA.) kits. Afterwards, reverse transcription of 1 μg of total RNA to complementary DNA was performed using the Superscript kit (Invitrogen, Carlsbad, CA, USA). All kits were used according with the manufacturer’s instructions. The expression levels of genes coding for MSC surface markers (CD90, CD105, CD73 and CD166), haematopoietic markers (CD34 and CD45) and immunogenic molecules (MHC-I and MHC-II) were analyzed using a StepOne Real Time PCR System device (Applied Biosystems) as described by Ranera et al. [31, 42]. All reactions were carried out in triplicate in a total volume of 10 μl with 2 μl of cDNA as template and Fast SYBR Green Master Mix (Applied Biosystems). The amplification consisted of 40 cycles of 3 s at 95 °C and 30 s at 60 °C. For analyzing and presenting the gene expression data, a relative expression method was used: the comparative Ct method was used to give the levels of gene expression. Expression level of every gene in each sample was normalized through a normalization factor (NF), calculated as the geometric mean of the quantity of 2 housekeeping genes, GAPDH and B2M [43]. The data of the gene of interest is presented as mRNA expression level relative to housekeeping genes, using the cycle threshold (Ct) values to calculate the expression.

Primer Express 2.0 software was used to design primers based on known equine sequences. Information about primers is shown in Additional file 4.

Proliferation rates, i.e. cell doubling times (DT), was calculated before freezing and after thawing using the formulae CD = ln (Nf/Ni)/ln2 and DT = CT/CD, where Nf indicates final number of cells; Ni indicates initial number of cells, CT indicates culture time and CD indicates cell doubling number.

For the experimental treatments, MSCs were detached from flasks by treating with 0.25 % trypsin-EDTA. MSCs were then rinsed three times with PBS and subsequently, three times with Lactated Ringer’s Solution in order to remove any residuary FBS.

### Clinical determinations

Evidence of synovial effusion and joint distention were visually assessed. One of the classical signs of inflammation is heat, which is exchanged between injured tissues and the environment [44]. Heat was quantified by measuring the skin temperature at each time point by non-contact infrared laser thermometer (GIM 530 MS Intelligent Multi Purpose Infrared Thermometer, Thermolab). Static and dynamic lameness examinations were performed following the parameters established by the American Association of Equine Practitioners (AAEP) [45]. Ultrasonography measurements were carried out in triplicate, using the same anatomical references. The evaluations of the articular surface, the thickness between skin and articular surface and the relaxation of the synovial recesses were performed using an Ultrasound machine (HDI-3500, ATL) and a 10 MHz linear transducer. All the parameters in the clinical assessment were evaluated by a researcher blinded to the Injections administered.

### Assessment of synovial fluids

Synovial fluid (2 mL) samples from each control and treated joints were taken at the predefined time points. Total protein values (TP) were measured by refractometry [46] using a RHB-32 Hand-held brix refractometer (Spectrum Technologies). White blood cells (WBC/μL) and neutrophil (NC/μL) countings were carried out using the *Neubauer* chamber. Joints with synovial values of TP <2 g/dL, WBC <1500 cells/μL and NC 250 neutrophil/μL were considered as healthy [47]. All the parameters assessed in the synovial fluid were evaluated by a researcher blinded to the Injections administered.

### Statistical analysis

Statistical analysis was performed using the SPSS 15.0. Differences between control and treated joints at each time point were analyzed by Student’s *t* test. Significance level was set at *P* < 0.05 for all analyses.

## Availability of supporting data

Supplementary data associated with this article can be found, in the online version, at doi:

## Additional files

Additional file 1: BM-MSC characterization data, part 2. BM-MSCs used in this studied were differentiated to adipogenic (A), osteogenic (B) and chondrogenic (C) lineages. Negative controls (no differentiation induction) for each differentiation assay are presented below the differentiation data from each lineage. (TIF 1300 kb) Additional file 2: Lameness score data. Lameness scores for each animal at each time-point. Both control limb (receiving LRS as vehicle control) and MSC limb (receiving autologous or allogeneic MSCs) were evaluated within groups of Injections 1, 2 and 3. (DOCX 17.5 KB) Additional file 3: Values of clinical and synovial parameters. Mean ± standard deviation (SD) of local temperature and synovial fluid analysis for autologous (Injection 1), allogeneic (Injection 2) and repeat allogeneic (Injection 3) mesenchymal stem cell (MSC) injected joints and their controls. Numeric values for local temperature (°C), total protein concentration (g/dL), White blood cell count (cells/μl) and neutrophil count (cells/μl) at each time point for Injection 1, Injection 2 and Injection 3 are provided. Asterisk symbol (*) indicates statistically significant difference (*p* <0.05) between control and MSC treated joints at each time point. (DOCX 18 kb) Additional file 4: Data of primers used for RT-qPCR. Cell surface markers analyzed by RT-qPCR. GenBank accession numbers of the sequences used for primers design. Primers (F: Forward and R: Reverse) and length of the amplicon in base pair (bp). (DOCX 18.0 KB)
