# Supplementary figures and images for: Inflammatory response to the administration of mesenchymal stem cells in an equine experimental model: effect of autologous, and single and repeat doses of pooled allogeneic cells in healthy joints
Source: BMC Vet Res. 2016 Mar 31;12:65. doi: 10.1186/s12917-016-0692-x (PMC4815220; doi:10.1186/s12917-016-0692-x)

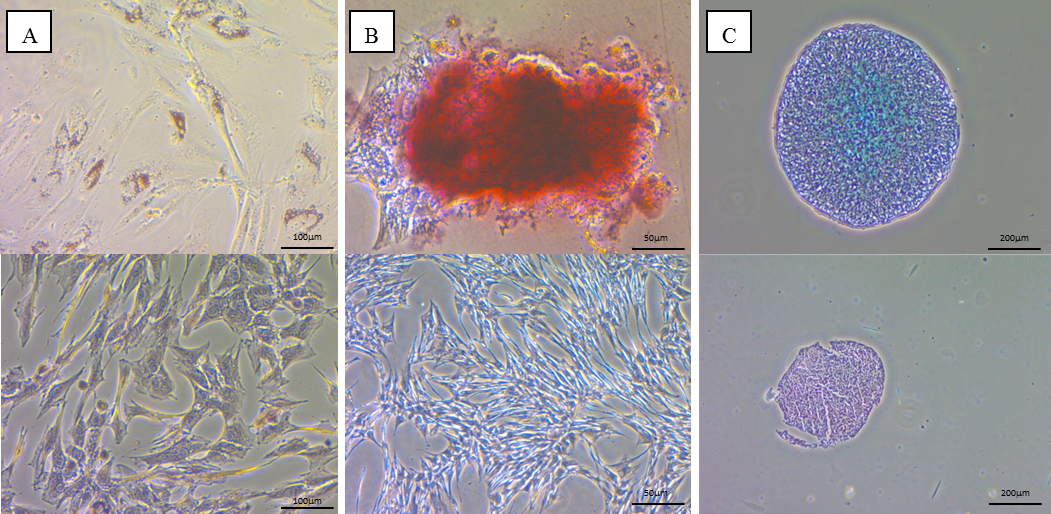

Supplement: Additional file 1: — BM-MSC characterization data, part 2. BM-MSCs used in this studied were differentiated to adipogenic (A), osteogenic (B) and chondrogenic (C) lineages. Negative controls (no differentiation induction) for each differentiation assay are presented below the differentiation data from each lineage. (TIF 1300 kb) [file 12917_2016_692_MOESM1_ESM.tif]
