# Supplementary material for: Inflammatory response to the administration of mesenchymal stem cells in an equine experimental model: effect of autologous, and single and repeat doses of pooled allogeneic cells in healthy joints
Source: BMC Vet Res. 2016 Mar 31;12:65. doi: 10.1186/s12917-016-0692-x (PMC4815220; doi:10.1186/s12917-016-0692-x)
Supplement: Additional file 2: — Lameness score data. Lameness scores for each animal at each time-point. Both control limb (receiving LRS as vehicle control) and MSC limb (receiving autologous or allogeneic MSCs) were evaluated within groups of Injections 1, 2 and 3. (DOCX 17.5 KB) [file 12917_2016_692_MOESM2_ESM.docx]

| EXPERIMENT | | 1 | | | | | | 2 | | | | | | 3 | | | | | |
| --- | --- | --- | --- | --- | --- | --- | --- | --- | --- | --- | --- | --- | --- | --- | --- | --- | --- | --- | --- |
| TIME (days) | | 0 | 1 | 2 | 3 | 5 | 10 | 0 | 1 | 2 | 3 | 5 | 10 | 0 | 1 | 2 | 3 | 5 | 10 |
| DIFFERENCE IN LOCAL TEMPERATURE (ºC) (TR. – CTRL) | CONTROL | 28,23 ± 0,52 | 28,34 ± 1,26 | 26,28 ± 0,50 | 26,18 ± 0,92 | 25,78 ± 0,62 | 30,60 ± 0,89 | 28,25 ± 5, 20 | 26,13 ± 6,74 | 24,57 ± 6,28 | 28,80 ± 3,02 | 31,07 ± 0,80 | 29,43 ± 2,36 | 32,43 ± 0,61 | 32,53 ± 1,22 | 32,83 ± 1,82 | 31,60 ± 2,73 | 32,93 ± 1,37 | 32,43 ± 0,92 |
|  | TREATED | 28,60 ± 0,57 | 30,38 ± 0,82 | 28,01 ± 0,15 | 25,90 ± 0,52 | 24,13 ± 0,80 | 30,85 ± 0,50 | 29,34 ± 3,73 | 26,50 ± 5,96 | 27,10 ± 5,08 | 30,02 ± 2,36 | 31,70 ± 0,17 | 30,07 ± 1,93 | 32,60 ± 0,51 | 32,97 ± 0,91 | 32,87 ± 1,78 | 32,57 ± 1,90 | 33,10 ± 1,03 | 32,70 ± 0,83 |
| TOTAL PROTEIN (g/dl) | CONTROL | 1,02 ± 0,16 | 1,90 ± 0,90 | 1,80 ± 0,69 | 1,67 ± 0,73 | 1,78 ± 0,92 | 1,18± 0,30 | 1,12 ± 0,26 | 1,90 ± 0,70 | 1,55 ± 0,56 | 1,50 ± 0,39 | 1,23 ± 0,23 | 0,63 ± 0,32 | 1,10 ± 0,35 | 0,83 ± 0,34 | 1,17 ± 0,61 | 1,07 ± 0,16 | 0,90 ± 0,30 | 0,83 ± 0,48 |
|  | TREATED | 1,07 ± 0,37 | 3,53 ± 0,81 * | 3,52 ± 0,90 * | 2,92 ± 1,18 | 2,40 ± 0,97 | 1,32 ± 0,40 | 1,35 ± 0,49 | 2,92 ± 1,68 | 2,88 ± 1,06  * | 2,27 ± 0,92 | 2,37 ± 0,63  * | 1,17 ± 0,56 | 1,13 ± 0,36 | 4,40 ± 1,09 * | 2,93 ± 0,55 * | 2,73 ± 0,82 * | 2,10 ± 0,68 * | 1,97 ± 1,47 |
| WHITE BLOOD CELL COUNT (cells/µl) | CONTROL | 307,50 ± 197,83 | 1989,17 ± 1985,43 | 1645,67 ± 2071,87 | 1145,17 ± 789,61 | 870,50 ± 579,24 | 387,50 ± 236,95 | 63,00 ± 31,01 | 2256,67 ± 4561,41 | 677,00 ± 905,28 | 3244,17 ± 3602,98 | 3299,50 ± 2536,03 | 1225,00 ± 1998,61 | 105,00 ± 106,16 | 141,67 ± 154,42 | 218,33 ± 253,94 | 1905,00 ± 2993,73 | 64,17 ± 44,09 | 250,83 ± 312,35 |
|  | TREATED | 98,33 ± 31,25 * | 29526,67 ± 25638,9 * | 16383,33 ± 10384,9 * | 13685,83 ± 13758,9 | 5370,00 ± 4241,98 * | 2486,33 ± 2353,0 | 271,00 ± 541,74 | 24035,00 ± 32778,3 | 17531,67 ± 19115,4 | 9393,33 ± 6988,65 | 6047,50 ± 6074,50 | 3021,67 ± 4121,18 | 452,50 ± 245,07 * | 29613,33 ± 17556,2 * | 8940,00 ± 5017,23 * | 4430,00 ± 2738,28 | 2140,00 ± 1563,1 * | 2140,00 ± 1563,1 |
| NEUTROPHILS COUNT (cells/µl) | CONTROL | 17,48 ± 26,67 | 475,28 ± 1004,96 | 87,29 ± 103,00 | 159,80 ± 302,98 | 175,89 ± 225,41 | 73,57 ± 103,22 | 3,30 ± 4,34 | 1022,47 ± 2111,86 | 41,93 ± 37,81 | 675,52 ± 936,02 | 245,42 ± 196,99 | 82,14 ± 141,09 | 20,32 ± 43,98 | 13,04 ± 15,28 | 79,58 ± 123,13 | 420,68 ± 989,64 | 3,50 ± 6,11 | 59,9 ± 125,40 |
|  | TREATED | 15,72 ± 28,60 | 22206,70 ± 22489,0 | 1948,79 ± 975,10 * | 3142,42 ± 3867,82 | 286,15 ± 287,12 | 87,12 ± 152,51 | 22,92 ± 56,13 | 12789,03 ± 22483,4 | 2311,58 ± 4105,41 | 706,98 ± 1009,58 | 188,72 ± 157,89 | 321,33 ± 768,54 | 43,28 ± 45,08 | 18127,20 ± 11162,2 * | 2013,20 ± 1620,25 * | 243,83 ± 394,11 | 115,23 ± 71,03 * | 111,71 ± 88,51 |

**Table S1**. Mean ± standard deviation (SD) synovial fluid analysis for autologous (experiment 1), allogeneic (experiment 2) and repeat allogeneic (experiment 3) mesenchymal stem cell (MSC) injected joints and their controls. Numeric values for difference in local temperature (ºC) between MSC injected joints (TR.) and control joints (CTRL.)**,** total protein concentration (g/dL), White blood cell count (cells/µl) and neutrophil count (cells/µl) at each time point for Experiment 1, Experiment 2 and Experiment 3 are provided. Asterisk symbol (*) indicates statistically significant difference (p <0.05) between control and MSC treated joints at each time point.
