# Supplementary material for: Inflammatory response to the administration of mesenchymal stem cells in an equine experimental model: effect of autologous, and single and repeat doses of pooled allogeneic cells in healthy joints
Source: BMC Vet Res. 2016 Mar 31;12:65. doi: 10.1186/s12917-016-0692-x (PMC4815220; doi:10.1186/s12917-016-0692-x)
Supplement: Additional file 4: — Data of primers used for RT-qPCR. Cell surface markers analyzed by RT-qPCR. GenBank accession numbers of the sequences used for primers design. Primers (F: Forward and R: Reverse) and length of the amplicon in base pair (bp). (DOCX 18.0 KB) [file 12917_2016_692_MOESM4_ESM.docx]

|  | INJECTION 1 | | | | | | | | | | | |
| --- | --- | --- | --- | --- | --- | --- | --- | --- | --- | --- | --- | --- |
| DAY | 0 | | 1 | | 2 | | 3 | | 5 | | 10 | |
| ANIMAL | Control limb | MSC limb | Control limb | MSC limb | Control limb | MSC limb | Control limb | MSC limb | Control limb | MSC limb | Control limb | MSC limb |
| 1 | 0 | 0 | 0 | 0 | 0 | 0 | 0 | 0 | 0 | 0 | 0 | 0 |
| 2 | 0 | 0 | 0 | 0 | 0 | 0 | 0 | 0 | 0 | 0 | 0 | 0 |
| 3 | 0 | 0 | 0 | 0 | 0 | 0 | 0 | 0 | 0 | 0 | 0 | 0 |
| 4 | 0 | 0 | 0 | 1 | 0 | 1 | 0 | 0.5 | 0 | 0.5 | 0 | 0 |
| 5 | 0 | 0 | 0 | 0 | 0 | 0 | 0 | 0 | 0 | 0 | 0 | 0 |
| 6 | 0 | 0 | 0 | 0 | 0 | 0 | 0 | 0 | 0 | 0 | 0 | 0 |
|  | INJECTION 2 | | | | | | | | | | | |
| DAY | 0 | | 1 | | 2 | | 3 | | 5 | | 10 | |
| ANIMAL | Control limb | MSC limb | Control limb | MSC limb | Control limb | MSC limb | Control limb | MSC limb | Control limb | MSC limb | Control limb | MSC limb |
| 1 | 0 | 0 | 0 | 0 | 0 | 0 | 0 | 0 | 0 | 0 | 0 | 0 |
| 2 | 0 | 0 | 0 | 0 | 0 | 0 | 0 | 0 | 0 | 0 | 0 | 0 |
| 3 | 0 | 0 | 0 | 0 | 0 | 0 | 0 | 0 | 0 | 0 | 0 | 0 |
| 4 | 0 | 0 | 0 | 0 | 0 | 0 | 0 | 0 | 0 | 0 | 0 | 0 |
| 5 | 0 | 0 | 0 | 0 | 0 | 0 | 0 | 0 | 0 | 0 | 0 | 0 |
| 6 | 0 | 0 | 0 | 0 | 0 | 0 | 0 | 0 | 0 | 0 | 0 | 0 |
|  | INJECTION 3 | | | | | | | | | | | |
| DAY | 0 | | 1 | | 2 | | 3 | | 5 | | 10 | |
| ANIMAL | Control limb | MSC limb | Control limb | MSC limb | Control limb | MSC limb | Control limb | MSC limb | Control limb | MSC limb | Control limb | MSC limb |
| 1 | 0 | 0 | 0 | 0 | 0 | 0 | 0 | 0 | 0 | 0 | 0 | 0 |
| 2 | 0 | 0 | 0 | 0 | 0 | 0 | 0 | 0 | 0 | 0 | 0 | 0 |
| 3 | 0 | 0 | 0 | 0 | 0 | 0 | 0 | 0 | 0 | 0 | 0 | 0 |
| 4 | 0 | 0 | 0 | 0 | 0 | 0 | 0 | 0 | 0 | 0 | 0 | 0 |
| 5 | 0 | 0 | 0 | 0 | 0 | 0 | 0 | 0 | 0 | 0 | 0 | 0 |
| 6 | 0 | 0 | 0 | 0 | 0 | 0 | 0 | 0 | 0 | 0 | 0 | 0 |
